# Overexpression of trefoil factor 3 (TFF3) contributes to the malignant progression in cervical cancer cells

**DOI:** 10.1186/s12935-016-0379-1

**Published:** 2017-01-05

**Authors:** Zhaohu Yuan, Dandan Chen, Xiaojie Chen, Huikuan Yang, Yaming Wei

**Affiliations:** 1 Department of Blood Transfusion, Guangzhou First People’s Hospital, Guangzhou Medical University, Guangzhou, 510180 Guangdong Province China; 2 Department of Radiology, Guangzhou First People’s Hospital, Guangzhou Medical University, Guangzhou, 510180 China

**Keywords:** Trefoil factor 3 (TFF3), Malignant progression, Cervical cancer cells, Therapeutic targets

## Abstract

**Background:**

There remains a great need for effective therapies for cervical cancers, the majority of which are aggressive leaving patients with poor prognosis.

**Methods and results:**

Here, we identify a novel candidate therapeutic target, trefoil factor 3 (TFF3) which overexpressed in cervical cancer cells and was associated with reduced postoperative survival. Functional studies demonstrated that TFF3 overexpression promoted the proliferation and invasion of cervical cancer cells, and inhibited the apoptosis by inducing the mRNA changes in SiHa and Hela cell lines. Conversely, TFF3 silencing disrupted the proliferation and invasion of cervical cancer cells, and induced the apoptosis via Click-iT EdU test, flow cytometry analysis and two-dimensional Matrigel Transwell analysis. Western blot analysis showed that overexpression of TFF3 repressed E-cadherin (CDH1) expression to promote the invasion of cervical cancer cells. Furthermore, down-regulated CDH1 via overexpression of TFF3 was significantly up-regulated by virtue of inhibitor of p-STAT3.

**Conclusions:**

These results suggested that TFF3 stimulated the invasion of cervical cancer cells probably by activating the STAT3/CDH1 signaling pathway. Furthermore, overexpression of TFF3 decreased the sensitivity of cervical cancer cells to etoposide by increasing P-glycoprotein (P-gp) functional activity. Overall, our work provides a preclinical proof that TFF3 not only contributes to the malignant progression of cervical cancers and but also is a potential therapeutic target.

**Electronic supplementary material:**

The online version of this article (doi:10.1186/s12935-016-0379-1) contains supplementary material, which is available to authorized users.

## Background

Worldwide, cervical cancer is ranked as the second most common cancer in women and the third leading cause of death from cancer in women [1, 2]. The incidence of cervical cancer is very high in developing countries [3]. Until recently, therapeutic options for hysterectomy-resistant cervical cancers have been limited with treatments largely palliative [4]. Therefore, detecting or preventing cervical cancers with progressions in early is critical, which could help to prolong patient survival. As we know TFF3 is a soluble peptide containing trefoil domain and C-terminal dimerization domain which is not only a novel prognostic marker but also a therapeutic target in various cancers, such as mammary carcinoma, gastric cancer and prostate carcinoma [5–8]. Upregulation of TFF3 after rectal cancer chemo-radiotherapy is an adverse prognostic factor [9]. Furthermore, in prostate carcinoma cells, TFF3 reduces the sensitivity to ionizing-radiation [10].

TFF3, behaved as an oncogene, promotes proliferation and invasion, improves survival, and increases oncogenicity in cancer cells, such as mammary carcinoma, gastric cancer and prostate carcinoma [5, 11]. TFF3 promoted epithelial tumorigenesis by inducing aberrant proliferation and inhibiting apoptosis [7]. TFF3 also may contribute to cancer metastasis with epithelial-to-mesenchymal transition (EMT) potentially through the regulation of genes such as androgen receptor (AR), FOXA1 and human epidermal growth factor receptor-type 2 (HER2) [12, 13]. Moreover, TFF3, a secreted protein, is a valuable predictive serum biomarker in patients with metastatic colorectal cancer [9]. In cancer cells, TFF3 promotes cell migration, invasion and metastasis by reducing cell–cell and cell–matrix interactions and enhancing cell scattering in bronchiole or other epithelia cells [14, 15]. Up-regulation of TFF3 in cancer cells was accompanied by activation of multiple pathways including PI3K, MAPK and JAK/STAT pathways which were associated with cellular proliferation, apoptosis, migration, invasion and clonogenic survival [16]. Despite the evidence that TFF3 could influence various cancer cells function in vitro, the role of TFF3 in cervical cancer cells has not been examined.

In the present study, we found that TFF3 protein was overexpressed in cervical cancer cells and weakly expressed in human non-tumor keratinocytes. We detected up-regulated expression of TFF3 promoted growth, proliferation and invasion, and inhibited apoptosis in SiHa and Hela cells. These finding demonstrate that TFF3 may be a potential therapeutic target in invasive cervical cancers with multidrug resistance.

## Methods

### Materials

Dulbecco’s modified Eagle’s medium (DMEM) and fetal bovine serum (FBS) were obtained from GIBCO (Carlsbad, California, USA). Mouse anti-GPADH polyclonal antibody (Lot#ab37168), Rabbit anti-Trefoil Factor 3 monoclonal antibody (Lot#ab108599), Mouse anti-E Cadherin monoclonal antibody (Lot#ab1416), Mouse anti-Phospho-STAT3 monoclonal antibody (Lot#ab119672), Mouse anti-Total STAT3 monoclonal antibody (Lot#ab119672) were obtained from Abcam (Cambridge, UK). JSI-124 was obtained from Enzo Life Science (USA). Goat anti-Rabbit IgG IR Dye 800cw (Lot#C30626-03) and Goat anti-Mouse IgG IR Dye 800cw (Lot#C40528-02) were from Odyssey (Licor, USA). Click-iT Edu imaging kit and Live/Dead Bac Light Viability Kit for microscopy were from Invitrogen (Carlsbad, CA, USA).

### Cell cultures and transfection

Human cervical cancer cell lines SiHa, CaSki, Hela, Me180 and human non-tumor keratinocyte line HaCaT were obtained from Nanjing KeyGen Biotech Co, Ltd (Nanjing,China). The cells were cultured in Dulbecco’s modified Eagle’s medium (GIBCO, Carlsbad, California, USA) containing 10% FBS in a humidified atmosphere of 5% CO_2_ at 37 °C. Human TFF3 expression, TFF3 siRNA and CDH1 siRNA plasmid constructs have been previously described [7, 17]. Luciferase assays were performed as previously described [18]. Briefly, transfections were carried out in triplicate using 1 μg of the appropriate luciferase reporter construct and empty vector per transfection along with 0.1 μg of Renilla luciferase construct as control for transfection efficiency. Luciferase activities were assayed after 24 h of transfection using the Dual Luciferase Assay System (Promega Corp, Madison, WI, USA).

### RT-PCR and semi-quantitative RT-PCR

Total RNA was isolated from cells using Trizol plus RNA Purification system as previously described [19]. DNase I treatment, total RNA to complementary DNA, PCR, and qPCR assays were performed as previously described. Gene expression analysis was performed as previously described [20] and the sequence of the primers were described in Additional file 1: Table S1.

Total RNA was isolated using Trizol plus RNA Purification Kit (Invitrogen, Carlsbad, CA) as previously described [19]. Semi-quantitative RT-PCR was performed using a Super Script One Step RT-PCR kit (Invitrogen, Carlsbad, CA, USA). Sequences of the nucleotide primers for RT-PCR were: TFF3 5′-GCTGCCAGAGCGCTCTGCATG-3′ and 5′-AAGGTGCATTTCTGCTTCCTGCAG-3′ (35 cycles; wild-type cell lines); β-Actin, 5′-ATGATATCGCCGCGCTCG-3′ and 5′-CGCTCGGTGAGGATCTTCA-3′ (23 cycles). Amplified RT-PCR products were visualized on a 1.5% agarose gel.

### In vitro invasion analysis

An in vitro invasion assay was carried out to examine the invasion of cervical cancer cells, as previously described [21]. Briefly, 24-well Transwell units with 8 µm polycarbonate nucleopore filters (Corning, NY, USA) were coated with 0.1 mL 0.8 mg/mL Engelbreth Holm-Swarm sarcoma tumor extract (EHS Matrigel) at room temperature for 1 h to form a genuinely reconstituted basement membrane. Cervical cancer cells (5 × 10^4^ cells) were placed in the upper compartment and 500 µL DMEM culture medium containing 10% fetal calf serum was added to the lower compartment. The Transwell plates were incubated at 37 °C for 36 h in a humidified atmosphere with 5% CO_2_ and stained with 10% crystal violet. Invading cells were defined as cells that had degraded the Matrigel and moved into the lower surface of the membrane. The non-invading cells retained on the upper surface of the membrane were removed by a cotton swab.

### Click-iT EdU test

We performed the Click-iT Edu test to analyze the cervical cancer cell proliferation according to the manufacturer’s instructions. Cervical cancer cells were incubated with EDU for 12 h and then images were obtained to determine percentages of EdU-labeled cervical cancer cells.

### Western blot

Cervical cancer cells lysates were separated by SDS-PAGE, blotted onto nitrocellulose membranes, and probed with primary antibodies, followed by Goat anti-rabbit or mouse IgG IR Dye 800 cw (1:15,000), respectively. Images were obtained with an Odyssey Imager (LI-COR, Lincoln, NE, USA).

### Flow cytometry and analysis

Flow cytometry analysis was performed either on FACSCAN using Cell Quest software, or on MACS quant seven color analyzer. Data analysis was performed using Flow Jo software.

### Total cell number

1 × 10^5^ (SiHa and Hela) cells were seeded into 35 mm^2^ falcon tissue culture dish in monolayers in 10% serum media in quadruplicate. On indicated days, cells were trypsinised and the cell number was determined using a haemocytometer.

### Graphs and statistical analysis

All graphs were generated using Prism 4 (GraphPad Software, Inc., California, USA). Statistical significance was assessed by using an unpaired two-tailed Student’s t test (P < 0.05 was considered as significant) using SPSS 18.0 (SPSS, Inc., Chicago, IL, USA). Columns are the mean of triplicate experiments; bars ± SD. *P < 0.05, **P < 0.01.

## Results

### TFF3 is overexpressed in human cervical cancer cell lines

**Fig. 1:**
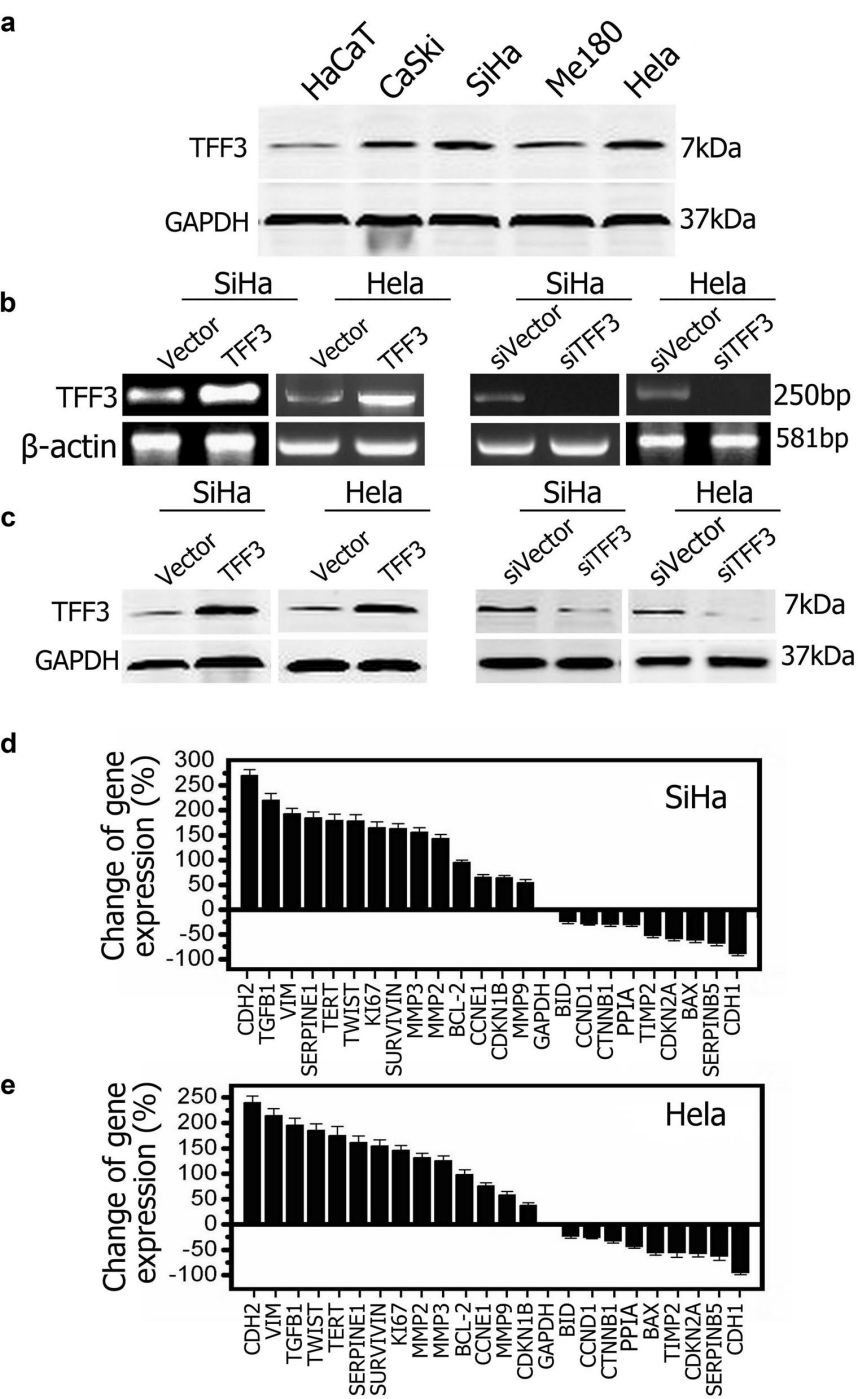
Forced expression of TFF3 in cervical cancer cells modulates the expression of malignant progression-related gene markers. **a** Determination of endogenous expression of TFF3 protein by Western blot in the cervical cancer cells lines CaSki, SiHa, Me180 and Hela and human non-tumor keratinocyte line HaCaT. **b** Semi-quantitative RT-PCR analysis was used to assess the mRNA levels of TFF3 in SiHa and Hela cells with either forced or depleted expression of TFF3 as described in “Methods” section. **c** Western blot analysis was used to assess the protein levels of TFF3 in SiHa and Hela cells with either forced or depleted expression of TFF3. **d**, **e** Quantitative PCR analysis quantifying the change in expression of various genes associated with malignant progression in SiHa-TFF3/Hela cells. Change in gene expression is expressed as fold difference, respectively. Fold change values are representative of three independent experiments

Compared with HaCaT cells, TFF3 was expressed at higher level in all the cervical cancer cell lines tested. To better reveal the functional roles of TFF3, SiHa and Hela cell lines were selected for next studies, in which expression of TFF3 was higher than the other two cervical cancer cell lines (Fig. 1a). To generate TFF3 overexpressing or knockdown stable cell lines, 293T cells were co-transfected with retroviral constructs of pMSCV-TFF3 or pSIH1-TFF3 along with the respectively package system plasmids for 2 days. The supernatant was collected to infect SiHa and Hela cell lines for 2 days, and then cells were collected for semi quantitative PCR and Western blot analysis (Fig. 1b, c).

Using RT-PCR analyses, we therefore quantified the mRNA levels of various molecules associated with cellular proliferation, apoptosis, migration, invasion and clonogenic survival in SiHa and Hela cell lines with forced expression of TFF3. SiHa-TFF3 or Hela-TFF3 cells exhibited decreased mRNA levels of BAX, TIMP2, CDKN2A, SERPINB5 and CDH1, relative to SiHa-vector cells or Hela-vector cells respectively. Concomitantly, SiHa-TFF3 and Hela-TFF3 cells exhibited increased mRNA levels of CDH2, VIM, TGFB1, TERT, SERPINE1, TWIST, KI67, SURVIVIN, MMP2 and MMP3, relative to SiHa-vector or Hela-vector cells (Fig. 1d, e). Considering the rapid changes in proliferative and molecular patterns of expression associated with the oncogenic characteristics, which indicated TFF3 plays an important role in the malignant progression of human cervical cancer.

### Overexpression of TFF3 promotes proliferation and survival of SiHa and Hela cells

**Fig. 2:**
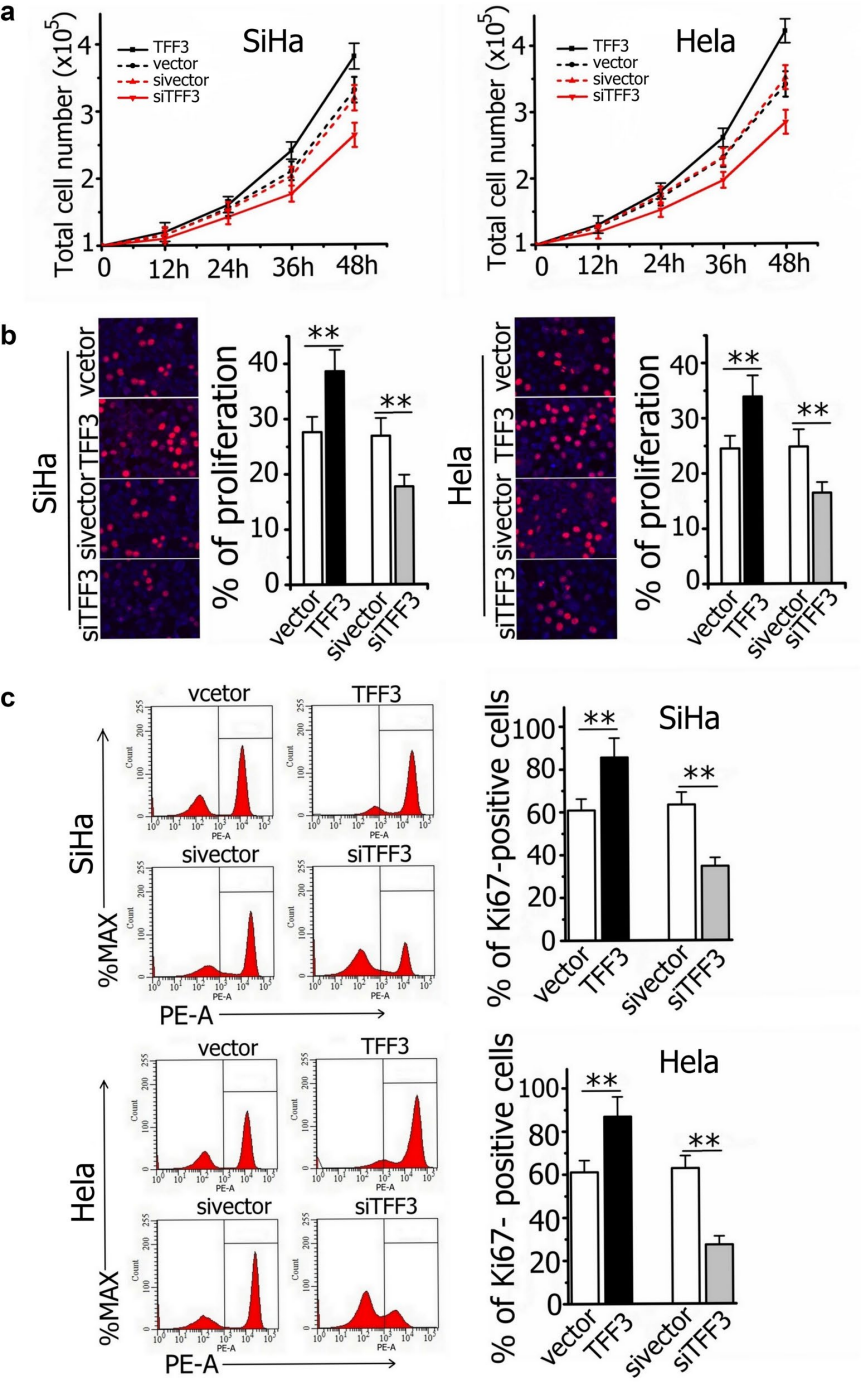
Correlation of TFF3 expression with the proliferation of SiHa and Hela cells. **a** Total cell number assays. Cells were seeded in both full-serum (10%) and total cell number counted every 12 h. **b** EdU incorporation and **c** Ki67 flow cytometry expression assays showed a statistically significant increase or decrease in proliferation when TFF3 was overexpressed or knockdown respectively in SiHa and Hela cells. Results represent mean ± SEM. **P < 0.01. Statistical significance was assessed by using an unpaired two-tailed Student’s test (*P* < 0.05 was considered as significant) using SPSS 18.0

To investigate whether TFF3 contributes to the proliferation of cervical cancer cells in vitro, we analyzed the proliferation upon overexpression and knockdown of TFF3 in SiHa and Hela cells. Overexpression of TFF3 increased the total cell number of SiHa and Hela cells by 1.20 fold and 1.26 fold respectively after 48 h. Conversely, knockdown of TFF3 decreased the total cell number of SiHa and Hela cells (Fig. 2a). Furthermore, TFF3 overexpressing cells (SiHa-TFF3 and Hela-TFF3 cells) showed a statistically significant increase in proliferation, as assessed by measuring EdU incorporation into DNA and Ki67 expression (Fig. 2b, c). In order to verify whether the endogenous function of TFF3 is to promote proliferation, it was followed by knocking down cervical cancer cells expressing high level of TFF3. Specific-stranded RNA oligonucleotides against TFF3 or negative RNA control were transfected into SiHa and Hela cells. When TFF3-specific oligonucleotides were used, a rapid down-regulation of TFF3 mRNA and protein was observed (Fig. 1b, c). The decrease in TFF3 level resulted in a concomitant decrease in proliferation. Cell proliferation assays (EdU incorporation and Ki67 expression) showed a statistically significant decrease in proliferation when TFF3 was down-regulated with siRNA (Fig. 2b, c).

**Fig. 3:**
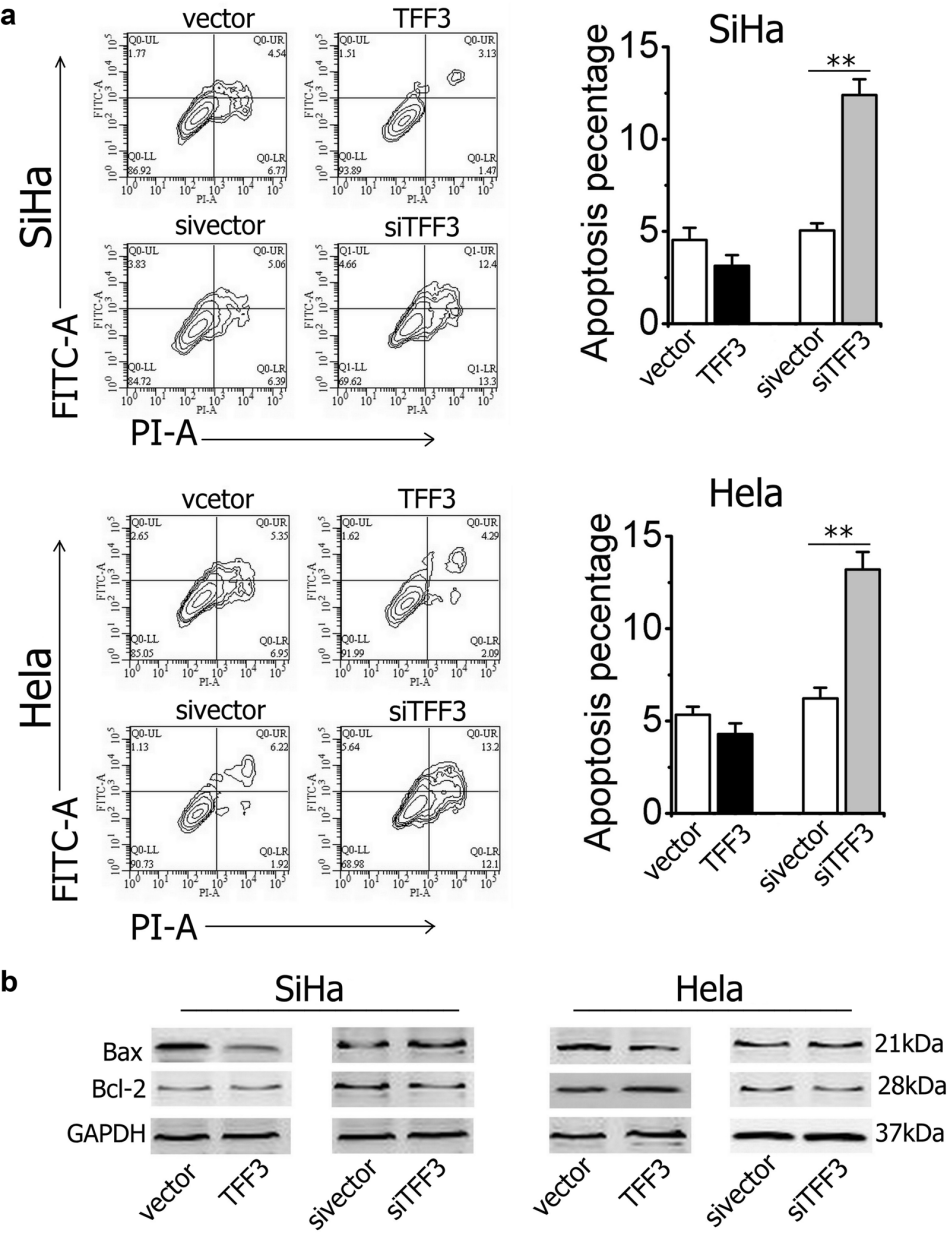
Effect of TFF3 on apoptosis of SiHa and Hela cell lines. **a** The flow cytometry analysis and quantification analysis of apoptotic cells. Statistical significance was assessed by using an unpaired two-tailed Student’s t test (*P* < 0.05 was considered as significant) using SPSS 18.0.* Columns* are the mean of triplicate experiments; *bars* ±SD. **P < 0.001. **b** Western blot analysis was used to assess the apoptosis related protein levels in SiHa and Hela cells with either forced or depleted expression of TFF3

The number of apoptosis cells was very low in TFF3-overexpressing cells (SiHa-TFF3 and Hela-TFF3 cells), but no significant differences from controls were observed (*P* > 0.05, Fig. 3a). However, the apoptotic proportions in SiHa-siTFF3 and Hela-siTFF3 were significantly increased (*P* < 0.01, Fig. 3a). Western Blot analysis demonstrated that forced expression of TFF3 reduced the levels of the Bax protein, whereas the Bcl-2 protein revealed only minimal increase in SiHa and Hela cells. The increase in Bcl-2/Bax ratio inhibits the apoptosis of human cervical cancer cells. Conversely, siRNA against TFF3 increased the expression of Bax protein and reduced the levels of Bcl-2 protein (Fig. 3b). Decreasing the rate of Bcl-2/Bax induces mitochondria-mediated apoptosis in human cervical cancer cells.

### CDH1 is critical for TFF3-mediated cervical cancer cells invasion

**Fig. 4:**
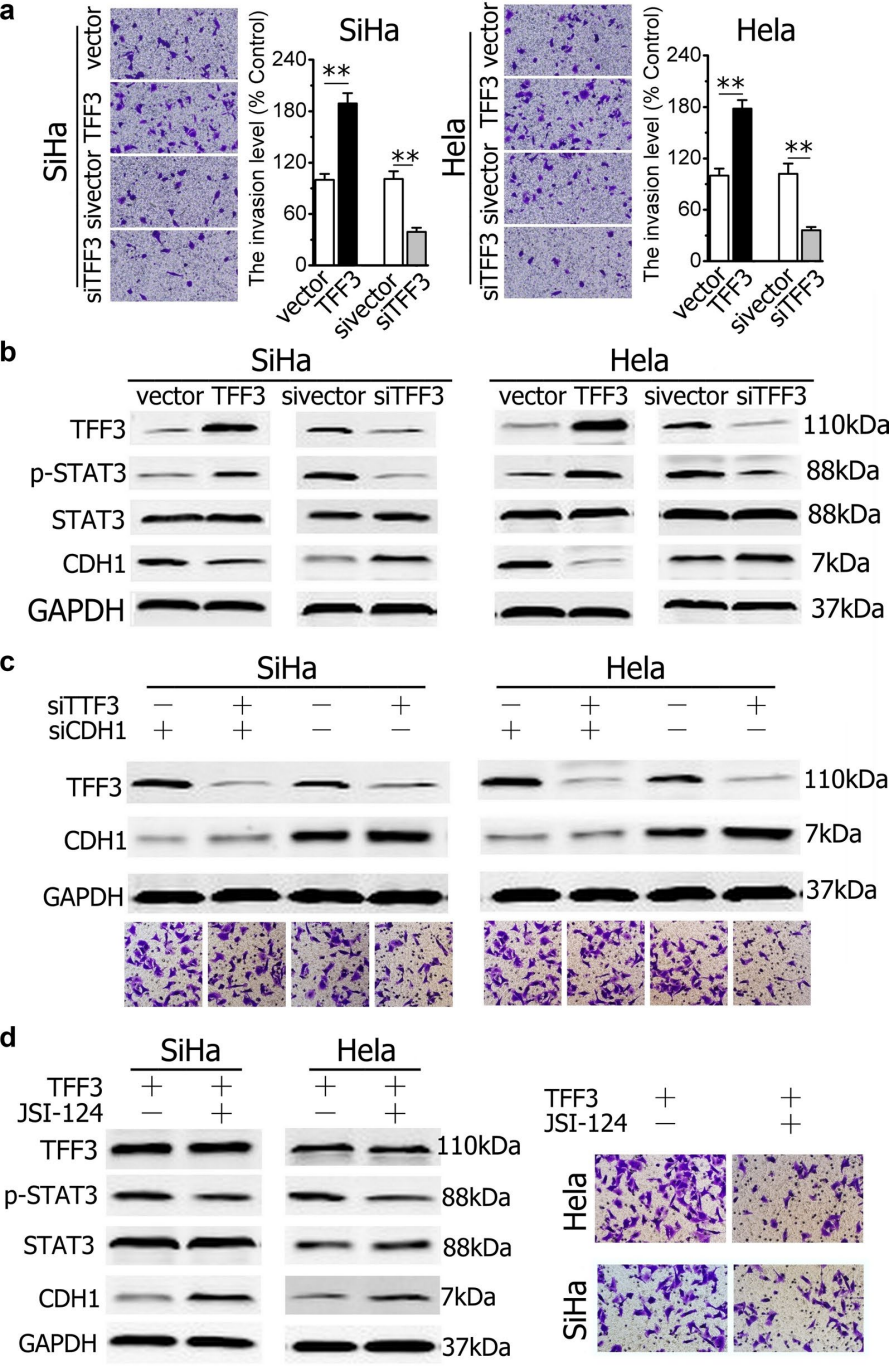
TFF3 regulates cell and invasion of cervical cancer cells through activation of the STAT3/CDH1 signaling pathway. **a** Capacity of SiHa and Hela cells with either forced or depleted expression of TFF3 to penetrate to EHS Matrigel and Quantification analysis. **b** Western blot analysis was used to assess the levels of CDH1, p-STAT3 andSTAT3 in cervical cancer cells with either forced or depleted expression of TFF3. **c** Effect of CDH1-specific siRNA on invasion of SIHA or HELA with depleted expression of TFF3. CDH1-specific siRNA increased the invasive percentage SiHa and Hela cells with depleted expression of TFF3. **d** Western blot analysis was used to assess the levels of CDH1 in SiHa or Hela cells with forced expression of TFF3on exposure to JSI-124 (0.2 μM) inhibitor and Effect of p-STAT3 on invasion of SiHa or Hela. Statistical significance was assessed by using an unpaired two-tailed Student’s t test (*P* < 0.05 was considered as significant) using SPSS 18.0. *Columns* are the mean of triplicate experiments; *bars* ±SD. **P < 0.001

Transwell invasion assays frequently were utilized to determine the invasiveness of cervical cancer cells in vitro. We therefore examined the behavior of cervical cancer cells with either forced or depleted expression of TFF3 growing on two-dimensional Matrigel. SiHa-TFF3 cells exhibited increased ability to penetrate and migrate in Matrigel-coated matrix compared with SiHa-vector cells (100.0 ± 7.2% for control cells vs. 189.5 ± 12.4% for TFF3-overexpressing cells; Fig. 4a). In contrast, the ability of SiHa-sivector to penetrate and migrate in Matrigel-coated matrix significantly decreased compared with SiHa-vector cells (101.3 ± 8.1% for control cells vs. 39.5 ± 4.2% for TTF3-knockdown cells; Fig. 4a). Similar results were obtained in Hela cells, as shown in Fig. 4a.

To further determine whether TFF3 could activate CDH1 expression in cervical cancer cells, TFF3-expressing plasmids were transiently transduced into the two cervical cancer cell lines. Western blot analysis was performed to examine the expression of TFF3 and CDH1. Consistent with the results reported previously [7], overexpression of TFF3 in the two cell lines suppressed expression of CDH1 and induced CDH1 signaling was evidenced by increasing phosphorylation of STAT3 in TFF3-overexpressing cells compared with control cells (Fig. 4b). Conversely, TFF3 knockdown in two cervical cancer cell lines increased CDH1 expression mRNA level (Fig. 1g, h) with concomitant decrease of phosphorylation of STAT3 (Fig. 4b). These data indicated that TFF3 could down-regulate CDH1 expression in cervical cancer cells.

Previous studies showed that overexpression of TFF3 promoted cell migration and invasion [6, 7, 11]. We next determined whether CDH1 mediated the effect of TFF3 on cell invasion in SiHa cells. We first utilized the CDH1-specific siRNA to knock down CDH1 expression in the two cell lines. Western blot analysis showed that CDH1 siRNAs achieved a knockdown of CDH1 expression in SiHa-siTFF3 cells by 70 to 80% (Fig. 4c). We performed cell invasion assay and found that the invasive ability in SiHa-siTFF3-siCDH1 cells was significantly stronger than that in SiHa-siTFF3 cells (Fig. 4c). In addition, transfection of CDH1 siRNA also increased invasive ability of control cells and the percentage was significantly greater compared with that in SiHa-siTFF3 cells (100.0 ± 9.1% for control cells vs. 142.1 ± 6.2% for TFF3-overexpressing cells; Fig. 4c). To further confirm that the STAT3/CDH1 signaling pathway is critical for TFF3-mediated cell migration and invasion, we treated TFF3-overexpressing or control cells with the STAT3 activity inhibitor, JSI-124. Western blots were used to confirm the blockage of the CDH1 signaling pathway. As expected, the phosphorylation level of STAT3 was decreased with JSI-124 treatment in TFF3-overexpressing cervical cancer cells (Fig. 4d). Moreover, the inhibition of STAT3/CDH1 pathway significantly attenuated cell invasion ability in TFF3-overexpressing cells (Fig. 4d). This is consistent with our hypothesis that overexpression of TFF3 could stimulate cell invasion through activation of the STAT3/CDH1 signaling pathway. Similar results also were obtained in Hela cells.

### TFF3 decreases the sensitivity of cervical cancer cells to etoposide by increasing P-gp functional activity

**Fig. 5:**
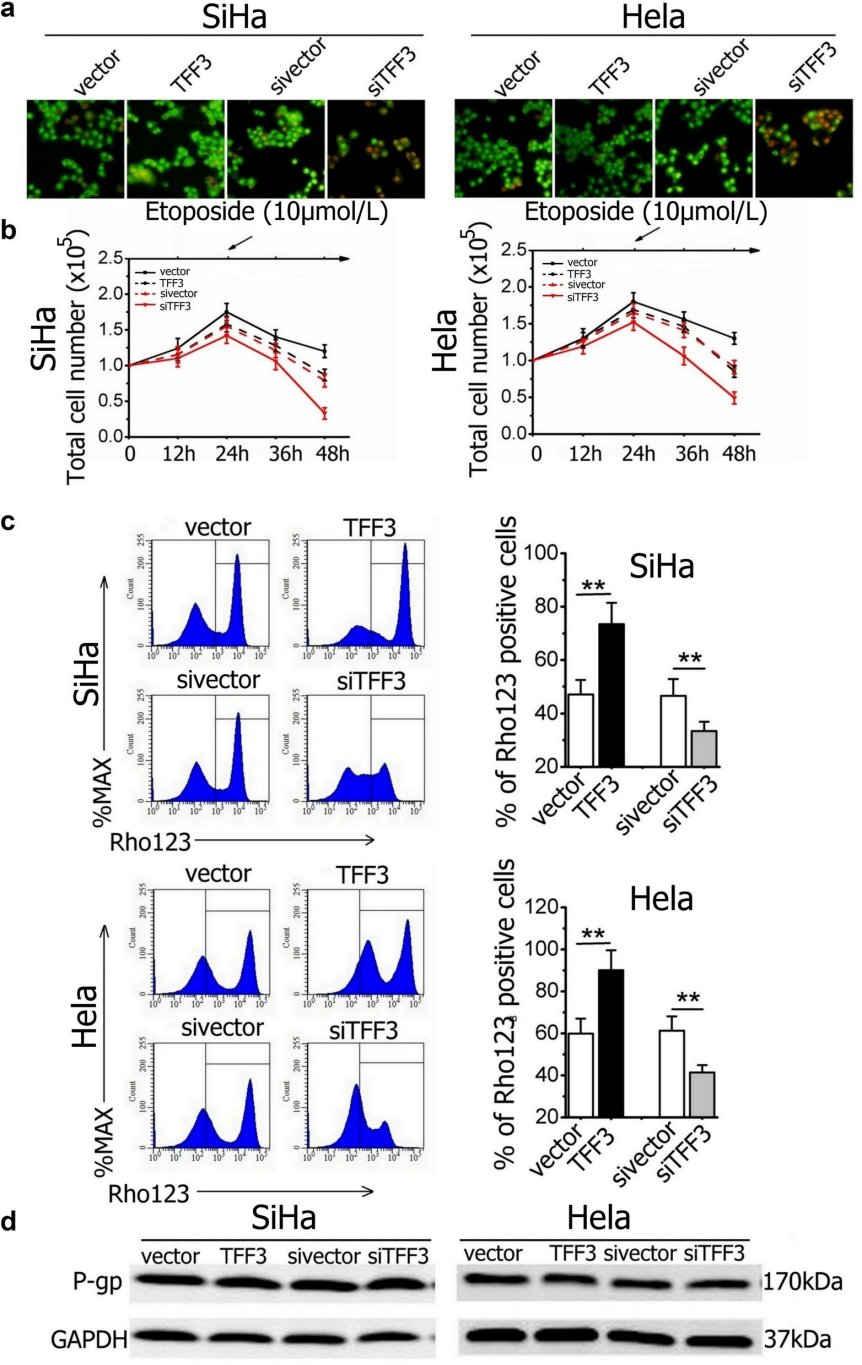
TFF3 decreased sensitivity of cervical cancer cells to etoposide by increasing P-gp functional activity (**a**) Cultures were stained with the live/dead stain. The cervical cancer cells were treated with 10 μmol/L etoposide for 24 h. Live cervical cancer cells with intact cell membranes stain fluorescent green, whereas cells with damaged membranes and dead cells stain fluorescent red (×200). **b** Total cell number assays of cervical cancer cells treated with etoposide (10 μmol/L) on 24 h. **c** The amount of Rho123 accumulated in cervical cancer cells was detected by flow cytometry to reflect P-gp function and Quantification analysis of Rho 123 positive cells. **d** The effect of TFF3 on expression of drug resistance-related protein, P-gp. Statistical significance was assessed by using an unpaired two-tailed Student’s t test (*P* < 0.05 was considered as significant) using SPSS 18.0. *Columns* are the mean of triplicate experiments; *bars* ±SD. **P < 0.001

MDR is a common clinical problem for the treatment of cancers in chemotherapy. Escaped cancer cells from chemotherapy through MDR are a major reason for clinical treatment failure [22, 23]. In this study we found forced expression of TFF3 in SiHa and Hela cells significantly decreased the sensitivity to etoposide and inhibited the apoptosis/death by live/dead staining. Conversely, TFF3 knockdown increased the sensitivity of cervical cancer cells to etoposide and promoted apoptosis (Fig. 5a, b).

Rho123 is a fluorescence substrate that is applied to investigate P-gp functional activity. When functional activity of P-gp declines, the accumulation of the Rho123 substrate within cells increases, and vice versa [24, 25]. Our results indicated that the amount of Rho123 accumulation in SiHa-TFF3 and Hela-TFF3 cells was significantly higher than that in control cells (47.06 ± 5.45% for control cells vs. 73.45 ± 8.01% for TFF3-overexpressing cells in SiHa cell line; 59.85 ± 7.17% for control cells vs. 90.14 ± 9.45% for TFF3-overexpressing cells in Hela; all *P* < 0.01; Fig. 5c). In contrast, the amount of Rho123 accumulated in SiHa-siTFF3 and Hela-siTFF3 cells significantly decreased compared with control cells (46.54 ± 6.32% for control cells vs. 33.44 ± 3.53% for TTF3-knockdown cells; 61.21 ± 6.84% for control cells vs. 41.43 ± 3.49% for TTF3-knockdown cells; all *P* < 0.01; Fig. 5c). However, western blot analysis showed that forced/deleted expression of TFF3 didn’t alter the expression level of P-gp. These results suggested that TFF3 increased intracellular accumulation of Rho123 by inhibiting P-gp pump function in SiHa and Hela cells and further decreased the sensitivity to etoposide.

## Discussion

This study contributes to our understanding of the molecular mechanism by which overexpression of TFF3 in human cervical cancers promotes tumor progression. The present work first found that TFF3 was overexpressed in cervical cancer cells and weakly expressed in human non-tumor keratinocytes. Several studies demonstrated that TFF3 overexpression strongly correlated with poor prognosis in various tumors [26, 27], which indicated that TFF3 could be a potentially superior diagnostic marker or therapeutic target for cervical cancer. In this study we demonstrated that TFF3 functionally promoted the malignant progression of cervical cancer cells. Forced expression of TFF3 promoted the proliferation and invasion, and inhibited the apoptosis in SiHa and Hela cells. Conversely, decreased expression of TFF3 inhibited the proliferation and invasion, and induced the apoptosis in the two cell lines. Our data suggested that TFF3 stimulated an invasive phenotype in cervical cancer cells through STAT3 mediated repression of CDH1. Furthermore, we found TFF3 decreased the sensitivity of cervical cancer cells to etoposide by increasing P-gp functional activity in the two cell lines. TFF3 silencing increased the sensitivity of the two cell lines to etoposide chemotherapy.

TFF3, behaved as an oncogene, promotes cancer cell proliferation, survival, oncogenicity and invasion in various cancers, such as mammary carcinoma, gastric cancer and prostate carcinoma [7–9]. For the first time, we found that TFF3 was overexpressed in cervical cancer cells. Elevated expression level of TFF3 has also been reported in the molecular apocrine subtype of estrogen receptor-negative mammary carcinoma characterized by the expression of AR, FOXA1 and a high frequency of HER2 expression [12, 13]. In SiHa and Hela cells, forced expression of TFF3 promoted cervical cancer cells growth, proliferation and invasion. Overexpression of TFF3 was caused changes in mRNA levels associated with the cellular proliferation, apoptosis, migration, invasion and clonogenic survival. Forced expression of TFF3 decreased mRNA expression of BAX, TIMP2, CDKN2A, SERPINB5 and CDH1, but increased mRNA levels of CDH2, VIM, TGFB1, TERT, SERPINE1, TWIST, KI67, SURVIVIN, MMP2 and MMP3 which closely correlated with increasing cell cycle progression, anti-apoptosis, proliferation, metastasis and invasion of cervical cancer cells [9, 10, 28, 29].

In the cervix cells, TFF3 expression was detected significantly higher level in cervical cancer cells than in human non-tumor keratinocytes. The results presented here clearly demonstrated that TFF3 overexpression accelerated cell cycle progression and a decrease in TFF3 levels slowed the progression of cells. In addition, TFF3 levels correlated with the proliferative potential of cervical cancer cells as revealed by correlation between TFF3 and Ki67 levels in vivo. As an oncogene, TFF3 is qualified with various functions that could impinge on normal cell proliferation. It is known that TFF3 induces the expressions of AR, FOXA1, HER2 and basic fibroblast growth factor (bFGF) in vitro in various cancers such as breast cancer and melanoma cells [12, 13, 30]. Our study revealed that overexpression of TFF3 had no effect on apoptosis in SiHa and Hela cells. But siRNA-mediated depletion of TFF3 induced the apoptosis of cervical cancer cells by decreasing anti-apoptotic protein, Bcl-2 and increasing pro-apoptotic protein, Bax. The ratio of anti-apoptotic and pro-apoptotic members within the Bcl-2 family plays an important role to determine cell fate [31–33].

It is well known that TFF3 is associated with invasion and metastasis which plays very important roles in progression of tumors. It was first discovered TFF3 might regulate migration via a Twist-dependent pathway in gastric cells [6], which was an indispensable step in the process of cell invasion. TFF3 participated in cancer invasion metastasis in breast cancer through repression of CDH1 mediated by STAT3 [7]. CDH1, as a tumor suppressor glyco-protein, is one of the major constituents of cell adhesion complexes and mediates calcium-dependent cellular interactions in epithelial cells, which plays a key role in the establishment of adherent type junctions [34, 35]. Loss of CDH1 expression, or CDH1 dysfunction, contributes to the loss of cell–cell interaction which stimulates cancer cells to gain an invasive cell phenotype and metastasis [36–38]. By two-dimensional Matrigel Transwell analysis we found TFF3 was critical for cervical cancers to involve invasion. Moreover, we observed that TFF3 repressed the expression of CDH1 to promote cell invasion in cervical cancer cells. Western blot analysis showed that siRNA against TFF3 increased the expression of CDH1 and decreased phosphorylation of STAT3 which up-regulated the expression of CDH1. Furthermore, up-regulated CDH1 via overexpression of TFF3 was significantly down-regulated by virtue of inhibitor of p-STAT3, JSI-124, which was similar to those reported by Pandey [7]. Our results suggested that TFF3 stimulates invasion of cervical cancer cells probably by, at least partially, activating the STAT3/CDH1 signaling pathway. CDH1 is a downstream protein of TFF3 and may be a key modulator of TFF3-mediated cervical cancer invasion.

Surgery combined with chemotherapy or radiotherapy is still the optimal treatment for cervical cancer while MDR causes the cervical cancer cells to be resistant to chemotherapeutic drugs resulting in chemotherapy failure [39–41]. Previously study showed that up-regulation of TFF3 after rectal cancer chemo-radiotherapy is an adverse prognostic factor. The physiological role of TFF3 in restoring the mucosa during neo-adjuvant chemotherapy could be interfering with treatment efficacy by increase neo-adjuvant chemotherapy resistance [9]. In this study, we showed that TFF3 decreased the sensitivity of cervical cancer cells to etoposide by increasing P-gp functional activity and had no effect on the expression of P-gp. Drug resistance to chemotherapy is mediated mainly by the overexpression of P-gp which is a phosphorylated transmembrane glycoprotein pump with ATP enzyme activity encoded by mdr-1 gene [42, 43]. P-gp exerts an impact on drug distribution by pumping a substantial amount of compounds from intracellular to extracellular sites, especially hydrated cation compounds. In addition, overexpression of TFF3 decreased the sensitivity of cancer cells to chemotherapy by mediating Bcl-2 [44, 45].

## Conclusions

In this study our evidence presented that TFF3 is expressed between human non-tumor keratinocyte line and cervical cancer cell lines distinctively, inducing proliferative activity and malignant progression of cervical cancer patients, strongly supporting a role for this gene in cervical carcinogenesis.

## Additional file

**Additional file 1: Table S1.** RT-PCR and semi-quantitative RT-PCR.

## Abbreviations

AR: androgen receptor
BAX: BCL2-associated X protein
CDH1: cadherin 1
CDH2: cadherin 2
CDKN2A: cyclin-dependent kinase inhibitor 2A
DMEM: Dulbecco’s modified Eagle’s medium
EMT: epithelial-to-mesenchymal transition
FBS: fetal bovine serum
FOXA1: forkhead box A1
HER2: human epidermal growth factor receptor-type 2
JAK: janus kinase
KI67: KI67 Antigen
MAPK: mitogen activated protein kinase-like protein
MDR: multidrug resistant
MMP2: matrix metallopeptidase 2
MMP3: matrix metallopeptidase 3
PI3K: PI3K phosphatidylinositol 3-kinase
P-gp: P-glycoprotein
SERPINE1: serpin peptidase inhibitor, clade E (nexin, plasminogen activator inhibitor type 1), member 1
SERPINB5: serpin peptidase inhibitor, clade B (ovalbumin), member 5
STAT3: signal transducer and activator of transcription 3
TERT: telomerase reverse transcriptase
TFF3: trefoil factor 3
TIMP2: TIMP metallopeptidase inhibitor 2
TGFB1: transforming growth factor beta 1
VIM: vimentin
