# Supplementary material for: Overexpression of trefoil factor 3 (TFF3) contributes to the malignant progression in cervical cancer cells
Source: Cancer Cell Int. 2017 Jan 5;17:7. doi: 10.1186/s12935-016-0379-1 (PMC5216547; doi:10.1186/s12935-016-0379-1)
Supplement: Supplementary file 1 — Additional file 1: Table S1. RT-PCR and semi-quantitative RT-PCR. [file 12935_2016_379_MOESM1_ESM.docx]

Table S1. qPCR primer sequence

| Gene | Sequences（5’→3’） | |
| --- | --- | --- |
| TFF3 | Sense | GCTGCCAGAGCGCTCTGCATG |
|  | Anti-sense | AAGGTGCATTTCTGCTTCCTGCAG |
| CDH2 | Sense | CACTGGTGGCACTACTAAG |
|  | Anti-sense | ACAATACAGAGGCAAAGC |
| TGFB1 | Sense | GCAACAATTCCTGGCGATAC |
|  | Anti-sense | CAACCACTGCCGCACAACT |
| VIM | Sense | CGGAGCCCGCTGAGACTTGA |
|  | Anti-sense | TGCTGTCCCGCCGATTGAGG |
| SERPINE1 | Sense | AATGCCCTCTACTTCAACG |
|  | Anti-sense | GTGCTGCCGTCTGATTTG |
| TERT | Sense | GGCGACATGGAGAACAAGC |
|  | Anti-sense | CGTGGGTGAGGTGAGGTGT |
| TWIST | Sense | TGAAAGGAAAGGCATCACTAT |
|  | Anti-sense | GGCCAGTTTGATCCCAGTA |
| KI67 | Sense | CAGCACCTGCTTGTTTGG |
|  | Anti-sense | TGGCTCCTGTTCACGTATTT |
| SURVIVIN | Sense | GACTTGGCCCAGTGTTTC |
|  | Anti-sense | CTCCCAAAGTGCTGGTATT |
| MMP3 | Sense | AAGGAGGCAGGCAAGACA |
|  | Anti-sense | CACGCACAGCAACAGTAGGA |
| MMP2 | Sense | TGGGAAATGTCAACAAGTATGA |
|  | Anti-sense | CTTCTGGCTGGGTCTGTG |
| BCL-2 | Sense | GAACTAAGGGTATGAAGGA |
|  | Anti-sense | CTGGAATCTAAAGGTCGT |
| CCNE1 | Sense | GGATGTTGACTGCCTTGA |
|  | Anti-sense | CACCACTGATACCCTGAAA |
| CDKN1B | Sense | CAGCTTGCCCGAGTTCTA |
|  | Anti-sense | ATGCGTGTCCTCAGAGTTAG |
| MMP9 | Sense | TCCCTGGAGACCTGAGAACC |
|  | Anti-sense | GCCACCCGAGTGTAACCAT |
| GAPDH | Sense | GGCACAGTCAAGGCTGAGAATG |
|  | Anti-sense | ATGGTGGTGAAGACGCCAGTA |
| BID | Sense | GTGAACCAGGAGTGAGTCGG |
|  | Anti-sense | GGAAGCCAAACACCAGTAGG |
| CCND1 | Sense | CTAGCAAGCTGCCGAACC |
|  | Anti-sense | TCCGAGCACAGGATGACC |
| CTNNB1 | Sense | GCAGCAACAGTCTTACCT |
|  | Anti-sense | ACAGGACTTGGGAGGTAT |
| PPIA | Sense | GTCCCAAAGACAGCAGAA |
|  | Anti-sense | GTGAAGTCACCACCCTGA |
| TIMP2 | Sense | GAGCACCACCCAGAAGAA |
|  | Anti-sense | CATCCAGAGGCACTCGTC |
| CDKN2A | Sense | GTGGACCTGGCTGAGGAGCT |
|  | Anti-sense | TGCGGGCATGGTTACTGC |
| SERPINB5 | Sense | GGCTTGGAGAAGATTGAA |
|  | Anti-sense | GAGTTTGACCTTGGCATT |
| CDH1 | Sense | CTGAGAACGAGGCTAACG |
|  | Anti-sense | GTCCACCATCATCATTCAATAT |
